# The Philosopher’s Stone the Subject of a Patent in the Fourteenth Century

**Published:** 1847-05

**Authors:** 


*The Philosopher's Stone the subject of a Patent in the Fourteenth Century.*—In a work lately published on the law of patents, it is stated "that in the time of Edward III. some alchymists persuaded the King that a philosopher's stone might be made, that the King granted a commission to two friars and two aldermen to inquire if it was feasible, who certified that it was, and that the King granted to the two aldermen a patent of privilege that they and their assigns should have the sole making of the philosopher's stone." This grant is believed to be the first, or at least the first patent privilege granted for any invention of which mention is made either in law books or chronicles, and is curious as showing the state of science in the fourteenth century.—*London Med. Gaz.*

